# Fe-oxide mineralogy of the Jiujiang red earth sediments and implications for Quaternary climate change, southern China

**DOI:** 10.1038/s41598-018-20119-4

**Published:** 2018-02-26

**Authors:** Ke Yin, Hanlie Hong, Thomas J. Algeo, Gordon Jock Churchman, Zhaohui Li, Zongmin Zhu, Qian Fang, Lulu Zhao, Chaowen Wang, Kaipeng Ji, Weidong Lei, Zhenggang Duan

**Affiliations:** 1 School of Earth Sciences, China University of Geosciences, Wuhan, Hubei 430074 China; 2 State Key Laboratory of Geobiology and Environmental Geology, the Ministry of Education, China University of Geosciences, Wuhan, Hubei 430074 China; 3 State Key Laboratory of Geological Processes and Mineral Resources, the Ministry of Education, China University of Geosciences, Wuhan, Hubei 430074 China; 4 Department of Geology, University of Cincinnati, Cincinnati, OH 45221-0013 USA; 5 School of Agriculture, Food and Wine, The University of Adelaide, South Australia, 5005 Australia; 6 Geosciences Department, University of Wisconsin – Parkside, Kenosha, WI 53141-2000 USA; 7 Gemological Institute, China University of Geosciences, Wuhan, Hubei 430074 China

## Abstract

Diffuse reflectance spectrophotometry (DRS) is a new, fast, and reliable method to characterize Fe-oxides in soils. The Fe-oxide mineralogy of the Jiujiang red earth sediments was investigated using DRS to investigate the climate evolution of southern China since the mid-Pleistocene. The DRS results show that hematite/(hematite + goethite) ratios [Hm/(Hm + Gt)] exhibit an upward decreasing trend within the Jiujiang section, suggesting a gradual climate change from warm and humid in the middle Pleistocene to cooler and drier in the late Pleistocene. Upsection trends toward higher (orthoclase + plagioclase)/quartz ratios [(Or + Pl)/Q] and magnetic susceptibility values (χ_lf_) support this inference, which accords with global climate trends at that time. However, higher-frequency climatic subcycles observed in loess sections of northern China are not evident in the Jiujiang records, indicating a relatively lower climate sensitivity of the red earth sediments in southern China.

## Introduction

Quaternary red earth sediments are widespread in the middle and lower reaches of the Yangtze River in southern China, and these deposits have experienced intense syndepositional pedogenesis under humid tropical to subtropical conditions^1,2^. Like the loess-paleosol sediments of northern China, the red earth sediments in southern China are also records of climatic and environmental changes during the period of their formation^3–7^.

**Figure 1:**
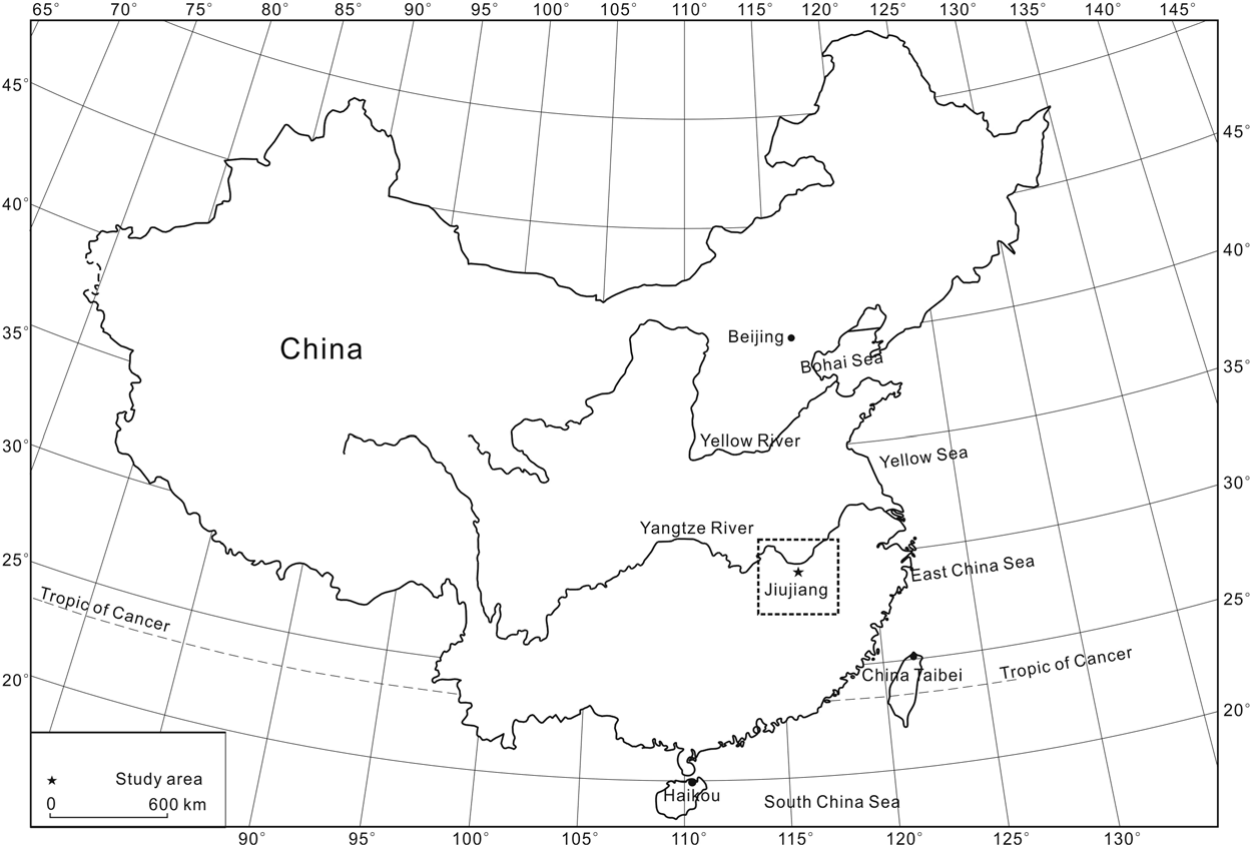
A generalized map showing the location of the study area. Adapted from a map of the National Administration of Surveying, Mapping and Geoinformation of the People’s Republic of China (http://bzdt.nasg.gov.cn/). The map is redrawn by Ke Yin using CorelDRAW X7.

**Figure 2:**
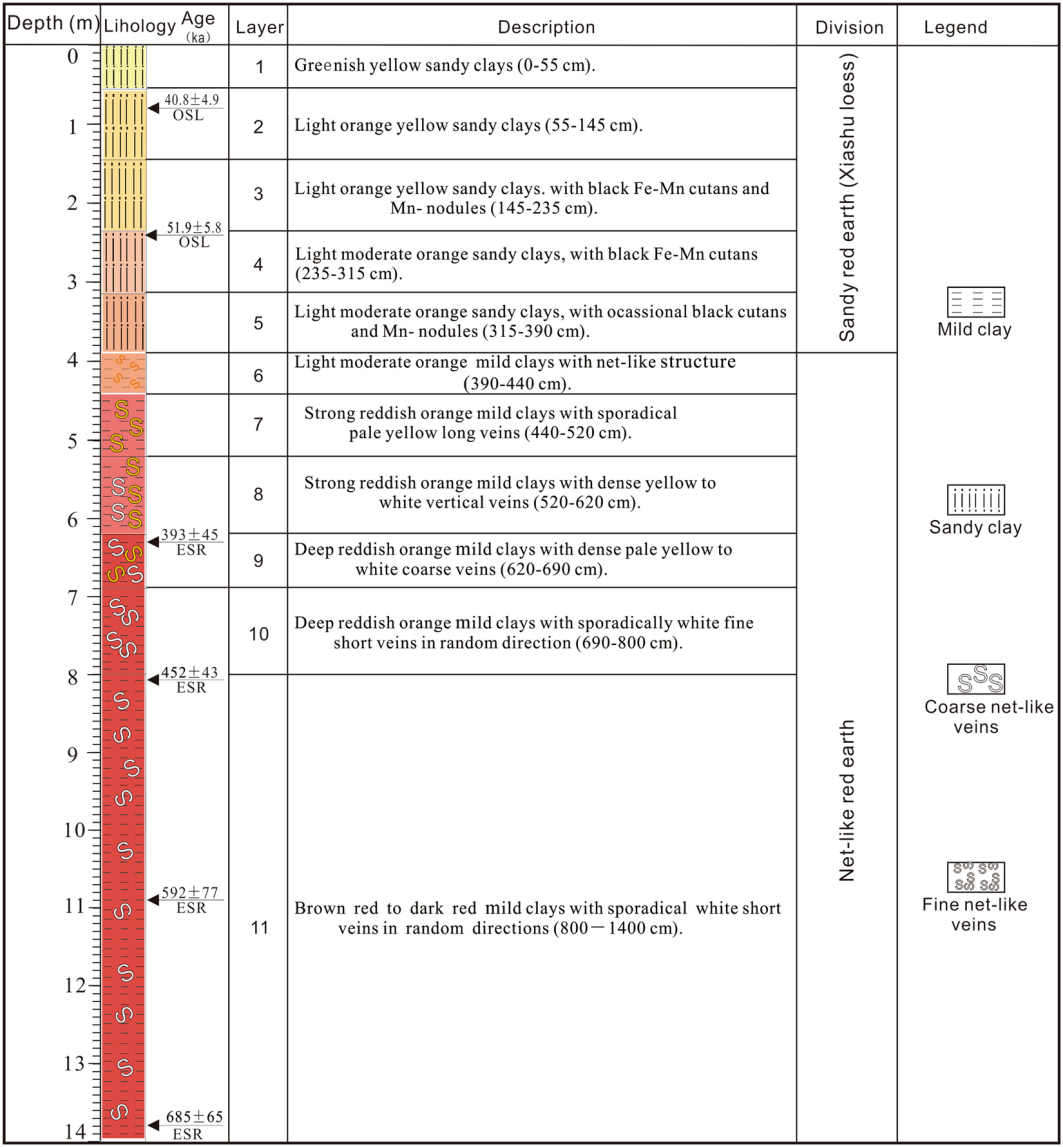
Stratigraphy of the Jiujiang section. OSL ages and ESR ages from Hong *et al*. (2013). Ke Yin created this figure using CorelDRAW14.

**Figure 3:**
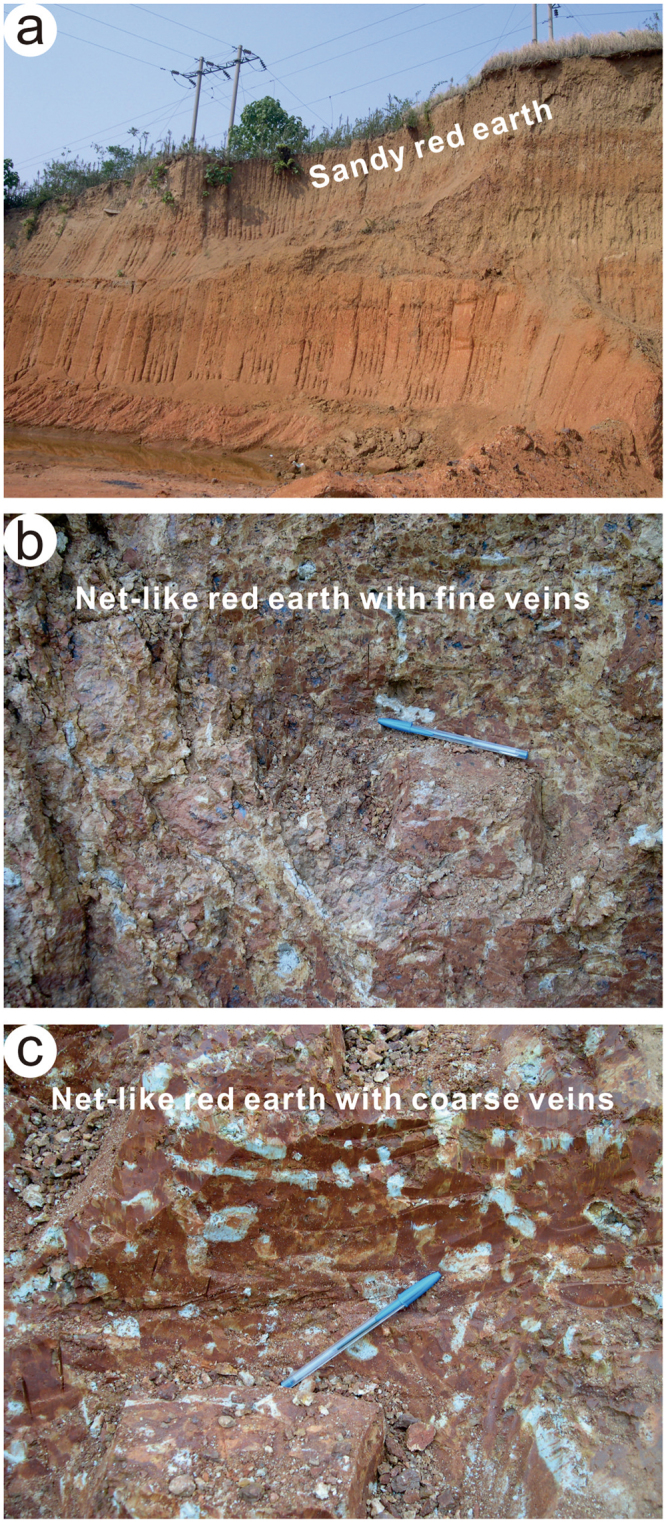
Photographs of the Jiujiang red earth section. Hanlie Hong took photo of the section, and Ke Yin edited these photos using CorelDRAW14. (**a**) Full view of section; (**b**) Close-up of middle unit, consisting of net-like red earth with fine veins; (**c**) Close-up of lower unit, consisting of net-like red earth with coarse veins.

The Jiujiang red earth section is located on the second terrace of the Yangtze River (Fig. 1), at 29°42′40.27″N, 116°00′13.7″E, 41 m above sea level^2^. The study area has a wet subtropical monsoonal climate that is warm and humid in summer and relatively cool and dry in winter, with mean annual precipitation (MAP) of 1000 to 1600 mm and mean annual temperatures (MAT) of 16–18 °C^2^. The red earth profile is 14 m thick and divided lithologically into three units (Fig. 2). The upper unit (0–3.9 m) consists of light orange yellow (7.5YR 9/8) to light moderate orange (2.5YR 7/8) sandy red earth, contains minor amounts of Fe-Mn cutans and Mn nodules, and exhibits a homogeneous texture without white veining. The middle unit (3.9–6.2 m) consists of light moderate orange (2.5YR 7/8) to strong reddish orange (7.5R 6/12) red earth and exhibits white fine net-like veins. The lower unit (6.2–14.0 m) consists of strong reddish orange (7.5R 6/12) to deep reddish orange (7.5R 4/12) red earth and exhibits white coarse net-like veins, which are more abundant and larger than those in the middle unit (Fig. 3). Optically stimulated luminescence (OSL) dating of samples at 0.8 and 2.2 m yielded ages of 40.8(±4.9) and 51.9(±5.8) ka, respectively, and electron spin resonance (ESR) dating of samples at 6.3, 8.1, 10.9, and 13.8 m yielded ages of 393(±45), 452(±43), 592(±77), and 685(±65) ka, respectively. The OSL method is usually more reliable for materials <100 ka and the ESR method more reliable for materials >100 ka. An age of 685(±65) ka for the base of the Jiujiang section indicates that the red earth accumulated after the middle Pleistocene^8^, and ages of 40.8(±4.9) and 51.9(±5.8) ka for the top of this section demonstrates that the upper unit was deposited during the late Pleistocene^9^. Average sedimentation rates are 12.6 cm/kyr for the upper unit (based on ages at 0.8 and 2.2 m) and 2.57 cm/kyr for the middle and lower units (based on ages at 6.3 and 13.8 m). Comparison with an earlier Sm-Nd isotopic study shows that the Jiujiang red earth sediments have a provenance similar to that of red earth deposits in the middle to lower reaches of the Yangtze River^10^. This suggests that all of the red earth sediments were generated within the middle to lower Yangtze River hydrologic basin.

After deposition, the Yangtze Basin red earth sediments underwent intense chemical weathering under warm and humid climatic conditions, as shown by high Rb/Sr ratios, intense depletion of mobile elements and concentration of immobile elements, as well as the well-developed net-like structure^10^. Although for the past few years the red earth sediments have attracted attention from many researchers in order to reconstruct paleoenvironments and paleoclimates in southern China, the climatic significance of these deposits is still debated. Studies of magnetic susceptibility and stable carbon isotopic compositions demonstrated the existence of eight depositional-pedogenic cycles in the Xiangyang section related to paleoclimatic changes^11–14^, although these findings were later challenged on the basis of an unreasonable degree of weathering of the “paleosols” and “loesses”^15,16^ and various soil parameters (Munsell color values, magnetic parameters, and stable carbon isotopic compositions)^17^. In view of these disagreements, further study of the southern Chinese red earth sediments is needed to evaluate the existence of multiple depositional-pedogenic cycles and their paleoclimatic significance.

Pedogenic processes associated with intense chemical weathering produce soil minerals reflecting the prevailing climatic conditions. Iron minerals, in particular, have proven to be powerful tools for reflecting soil formation intensity and paleoclimatic evolution^18,19^. Goethite and hematite, which have similar thermodynamic stabilities, are the most common Fe oxides in soils. Soil factors such Eh, pH, temperature, and organic matter content influence the formation and stability of goethite and hematite^19,20^. Cooler and wetter conditions promote formation of goethite, which imparts a yellow-brown color to soils, whereas warmer and drier conditions favor hematite^19^. Hematite, especially the fine-grained variety, imparts a reddish color to soils, that masks the yellowish color of goethite^21^. Therefore, the ratio of hematite to goethite (i.e., hematite/(hematite + goethite, or Hm/(Hm + Gt)) is commonly used as a climate proxy in soil studies^18,22^. However, traditional analytical techniques such as visually measured soil color proxies, X-ray diffraction, and Mössbauer spectrometry are either not sufficiently precise or too time-consuming for determination of hematite and goethite concentrations in large numbers of samples^22–24^. The recent development of diffuse reflectance spectroscopy (DRS) has provided a faster, more precise, and nondestructive method of quantification of hematite and goethite in soils^22,25^. The presence of either hematite or goethite can be detected at less than 0.1% in mixtures with other soil minerals^22,26^. Rock magnetic techniques can also be used to obtain information about pedogenic processes and paleoclimate evolution from iron-oxide assemblages in soils^27–32^. The most common pedogenic magnetic minerals in red earth sediments of southern China are maghemite, hematite, and goethite, and transformations among them result in variable mineralogic concentrations and magnetic intensities in soils^29^. Thus, a combination of rock magnetic and spectroscopic techniques can be used to retrieve paleoclimatic information recorded in the Fe-oxide fraction of paleosols^25,33^.

In response to climatic warming and increased humidity, the red earth sediments in southern China were subject to relatively intense pedogenic alteration^8^, resulting in soil-mineral transformations, especially among iron oxides. The Jiujiang red earth sediments were accretionary in nature, reflecting some degree of syndepositional pedogenesis under tropical to subtropical conditions^8^. Therefore, we take it that post-depositional diagenesis played a very weak role in the red earth. The main goal of the present study was to investigate the Fe-oxide mineralogy of the Jiujiang red earth section, with supplementary information from silicate minerals, in order to reveal the paleoclimatic evolution of the middle to lower Yangtze River of southern China during the middle and late Pleistocene.

## Results

### Diffuse reflectance spectrophotometry (DRS)

**Figure 4:**
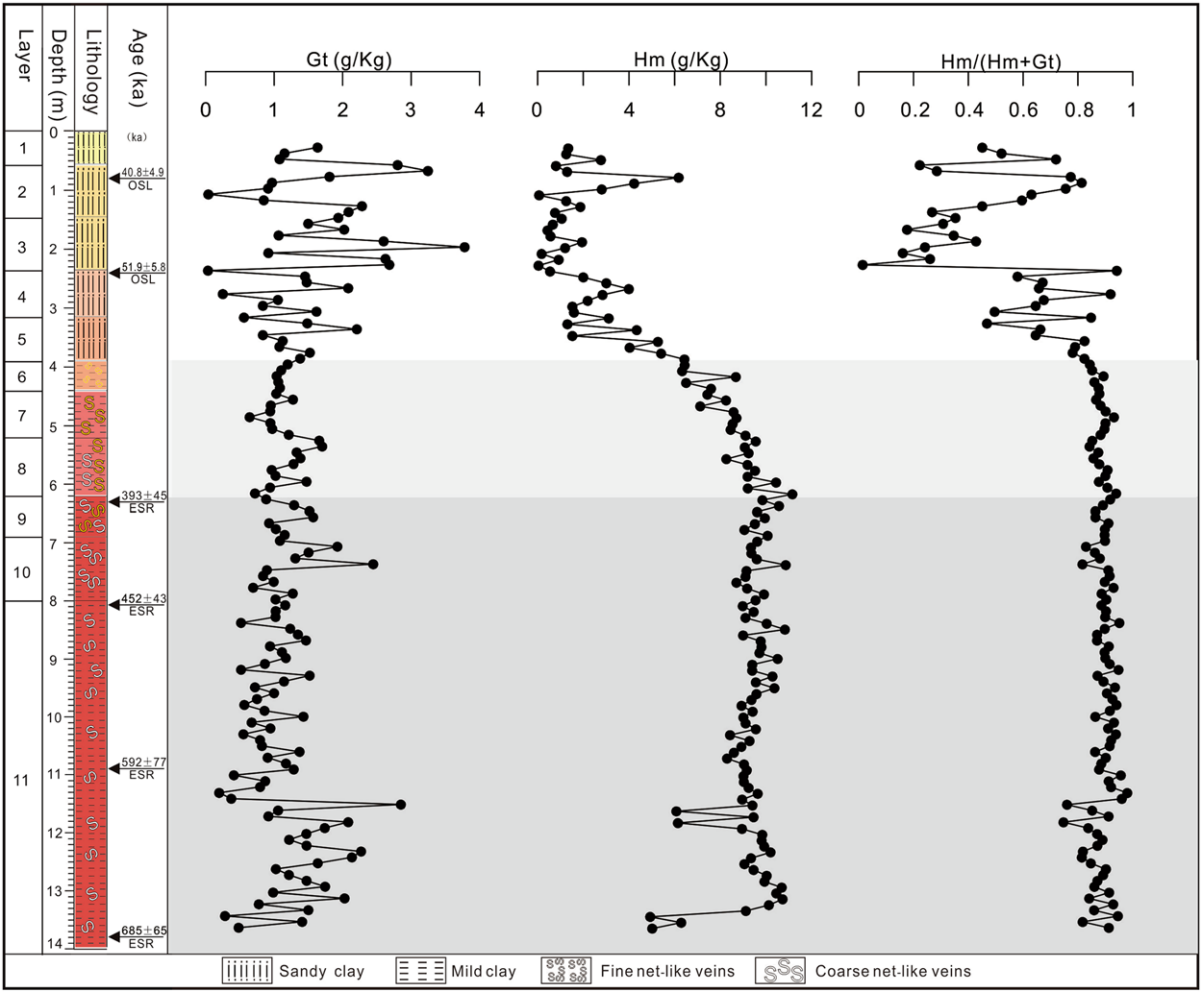
Variations of hematite and goethite content and Hm/(Hm + Gt) along the Jiujiang red earth section. Ke Yin created this figure using Grapher 9 and CorelDRAW14. a-δ^18^O records of the equatorial Pacific Core V28-238 adapted from Shackleton and Opdyke, 1973; b- magnetic susceptibility record of the Chinese loess obtained by averaging data from localities Luochuan and Xifeng (Kukla *et al*.^56^). (Luochuan County, Shanxi Province) and Xifeng (Xifeng District, Gansu Province) ~360 km to the west.

In the Jiujiang red earth section, hematite content ranges from 0.04 to 11.16 g kg^−1^, whereas goethite content ranges from 0.03 to 3.78 g kg^−1^. The Hm/(Hm + Gt) ratio ranges from 0.01 to 0.98. Based on these proxies, three stages of accumulation of the Jiujiang red earth section can be identified. The lower unit (6.2–14.0 m) contains the highest concentrations of hematite (4.93 to 10.87 g kg^−1^; mean 9.28 g kg^−1^) and the lowest concentrations of goethite (0.20 to 2.85 g kg^−1^; mean 1.16 g kg^−1^), yielding the highest Hm/(Hm + Gt) ratios (0.75 to 0.98; mean of 0.89). The hematite content in the lower unit (6.2–14.0 m) is relatively stable although with minor fluctuations at depths of 11.4–12.0 m and a larger negative excursion at depths of 13.0–14.0 m. Its goethite content ranges from 0.20 to 2.85 g kg^−1^, with relatively higher content at depths of 11.0–14.0 m (mean 1.27 g kg^−1^). The middle unit (3.9–6.2 m) also contains more hematite (6.33 to 11.6 g kg^−1^; mean 8.46 g kg^−1^) than goethite (0.64 to 1.70 g kg^−1^; mean 1.14 g kg^−1^), although Hm/(Hm + Gt) ratios (0.81 to 0.91; mean of 0.84) are lower than in the lower unit. Within the middle unit, both the hematite and goethite content decline upsection, although the decline in hematite is relatively greater, imparting a pale yellow to yellowish-brown color to the upper part of this unit. The upper unit (0–3.9 m) exhibits the lowest hematite concentrations (0.04 to 6.43 g kg^−1^; mean 2.18 g kg^−1^) and the highest goethite concentrations (0.03 to 3.78 g kg^−1^; mean of 1.54 g kg^−1^), yielding the lowest Hm/(Hm + Gt) ratios (0.01 to 0.94; mean of 0.51). The contents of both hematite and goethite oscillate considerably, and neither proxy exhibits an obvious decreasing or increasing trend upsection. For the Jiujiang section as a whole, goethite content fluctuates more strongly than hematite content, which is relatively stable in the lower and middle units and oscillates only in the upper unit. From the base of the section upward, goethite exhibits an overall increasing trend and hematite a decreasing trend resulting in a pronounced decline in Hm/(Hm + Gt) ratios upsection (Fig. 4).

### Magnetic susceptibility (χ_lf_)

**Figure 5:**
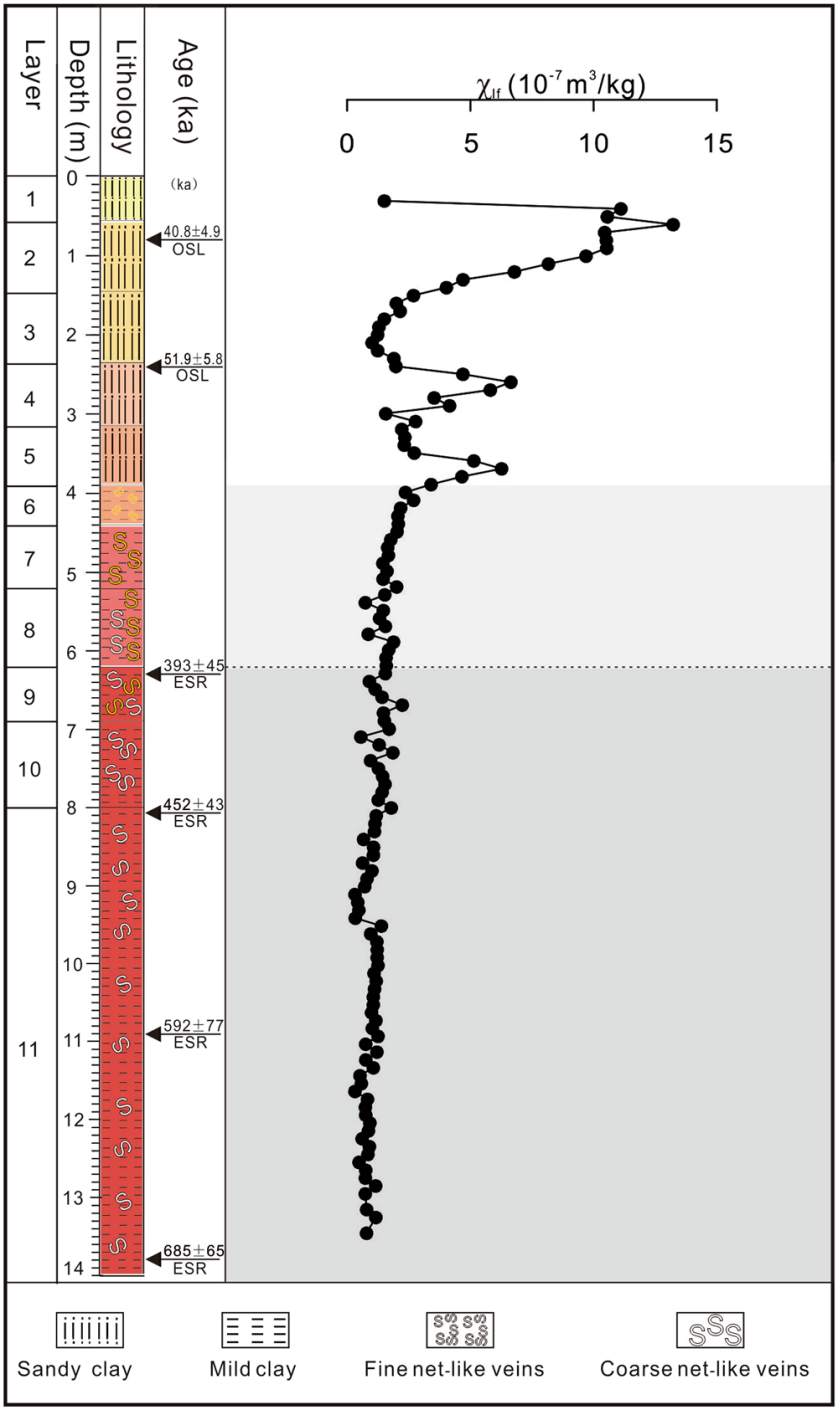
Low-field magnetic susceptibility of Jiujiang red earth section. Ke Yin created this figure using Grapher 9 and CorelDRAW14.

The magnetic susceptibility (χ_lf_) of the entire section ranges from 3.15 × 10^−8^ to 1.32 × 10^−6^ m^3^ kg^−1^ with a mean of 2.21 × 10^−7^m^3^ kg^−1^. χ_lf_ is most variable in the upper unit (1.01 × 10^−7^ to 1.32 × 10^−6^ m^3^ kg^−1^; mean 4.77 × 10^−7^ m^3^ kg^−1^), whereas it exhibits smaller fluctuations in the middle unit (7.34 × 10^−8^ to 2.70 × 10^−7^ m^3^ kg^−1^; mean of 1.70 × 10^−7^ m^3^ kg^−1^) and lower unit (3.15 × 10^−8^ to 2.23 × 10^−7^ m^3^ kg^−1^; mean of 1.02 × 10^−7^ m^3^ kg^−1^). χ_lf_ exhibits an overall increasing trend upsection (Fig. 5).

### X-ray diffraction (XRD)

**Figure 6:**
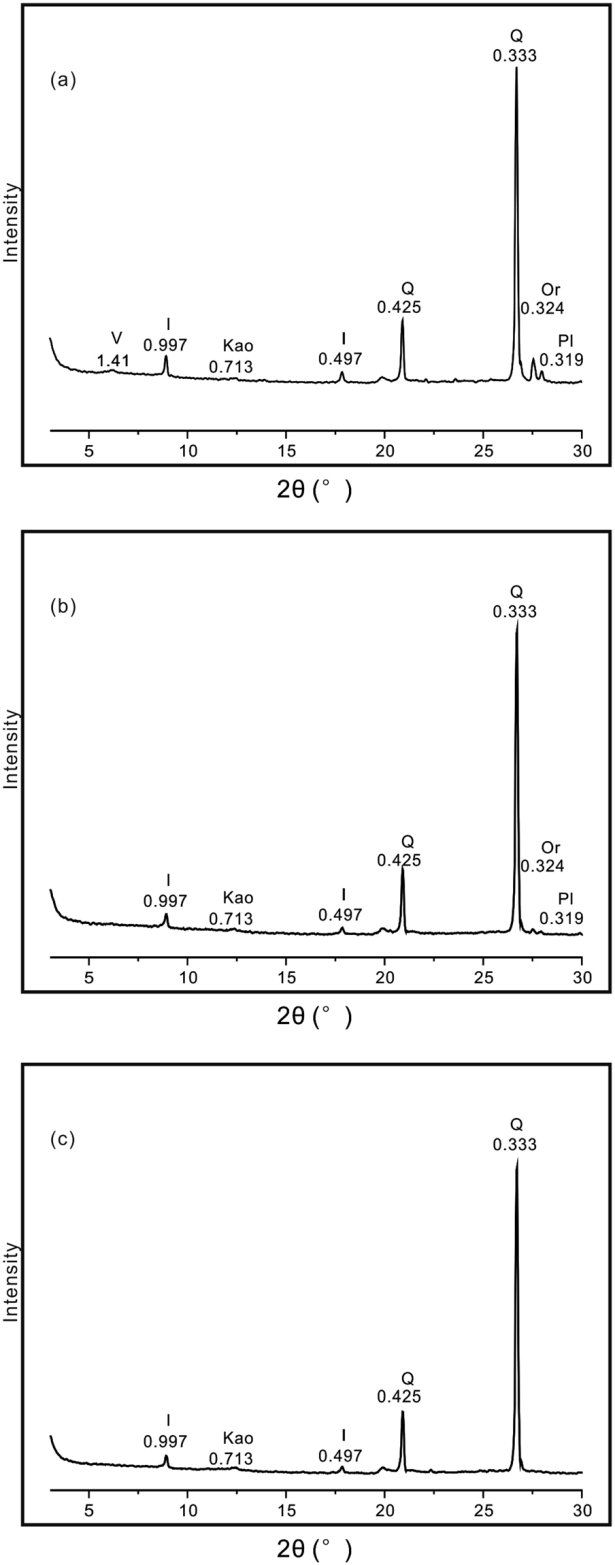
XRD patterns of representative bulk samples from the upper (**a**), middle (**b**), and lower (**c**) units of the Jiujiang red earth section. I-illite; Kao-kaolinite; Q-quartz; Or-orthoclase; Pl-plagioclase. Ke Yin created this figure using Grapher 9 and CorelDRAW14. I-illite; Kao-kaolinite; Q-quartz; Or-orthoclase; Pl-plagioclase.

**Figure 7:**
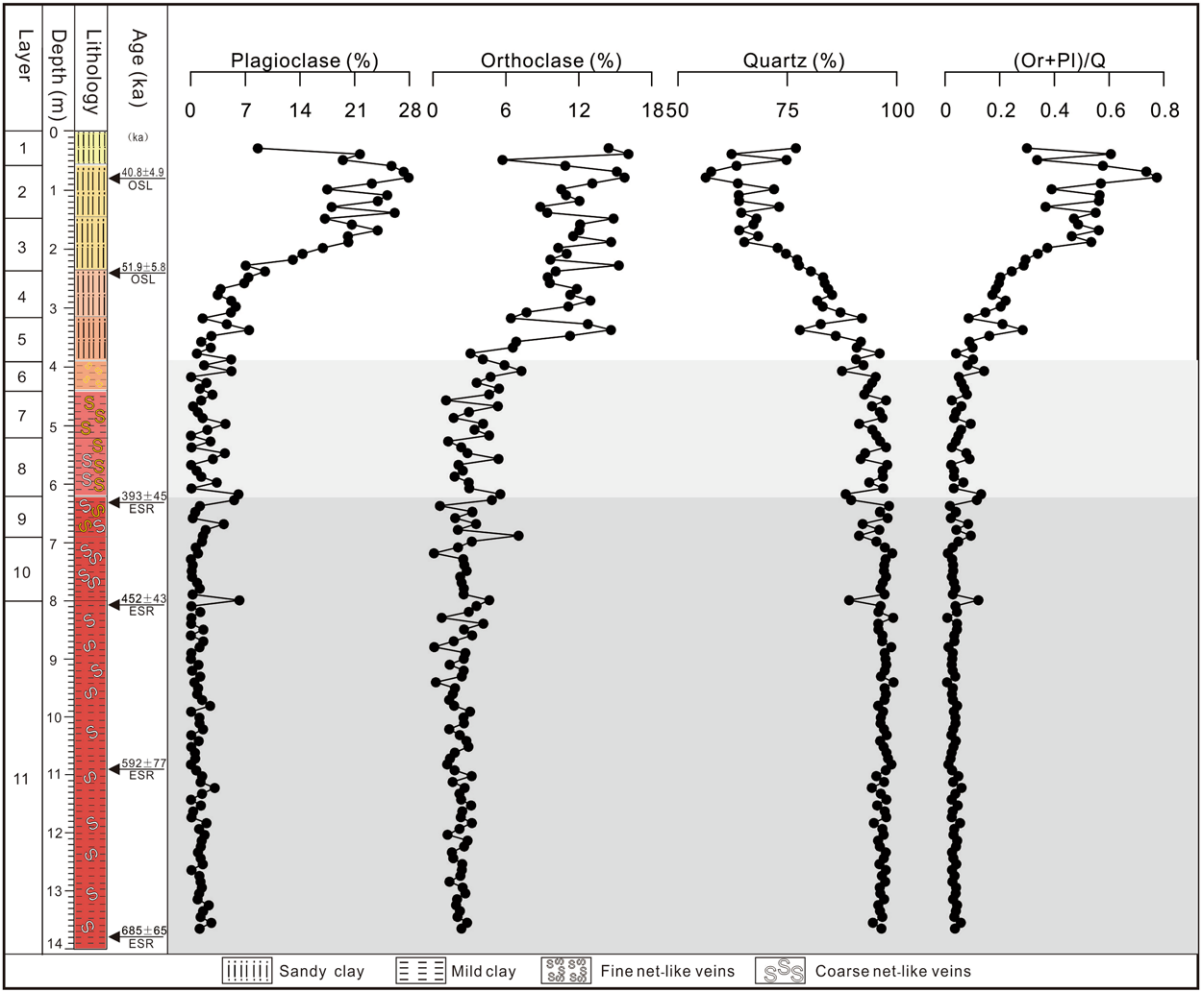
Plagioclase, orthoclase, and quartz content and Or/(Or + Pl) of Jiujiang red earth section. Ke Yin created this figure using Grapher 9 and CorelDRAW14.

X-ray diffraction analysis of the Jiujiang red earth section shows that all bulk samples contain similar clay assemblages and other mineral species. The main non-clay minerals are quartz (identified from the 0.425 and 0.333 nm peaks) and minor feldspars (orthoclase and plagioclase identified from the 0.324 and 0.319 nm peaks, respectively) (Fig. 6a,b,c). The absence of other silicates suggests intense chemical weathering conditions since the middle Pleistocene. Feldspars (both orthoclase and plagioclase) have notably higher contents in the upper unit than in the middle and lower units (Fig. 7), a pattern suggesting decreasing weathering intensity towards the surface of the profile.

The main clay minerals in the Jiujiang red earth section are illite, kaolinite, and vermiculite, with trace amounts of mixed-layer kaolinite–smectite and mixed-layer illite–vermiculite, as determined from oriented clay samples in previous XRD studies^8,34–36^. Bulk-sample XRD data show that the clay assemblages of the lower and middle units are dominated by illite and kaolinite, whereas the upper unit contains illite, vermiculite, and kaolinite. Under acidic soil-water conditions, high-charge vermiculite can transform into kaolinite via mixed-layer vermiculite-kaolinite^37–39^. However, the amounts of the two mixed-layer phases in the Jiujiang section are quite low, as shown by an absence of characteristic peaks in bulk-sample XRD traces. The absence of vermiculite in bulk samples from the middle and lower units is due to their more highly weathered condition compared to the upper unit, reflecting warmer and more humid climatic conditions during their formation. Feldspars and vermiculite were more stable under the less intense weathering conditions that prevailed during accumulation of the upper unit. Hence, the clay mineralogy of the Jiujiang red earth section records a major paleoclimate shift from warm/humid to cool/dry conditions during the middle to late Pleistocene.

## Discussion

Goethite is generally the dominant Fe-oxide in loess-paleosol sections of northern China owing to locally cool and wet pedoclimatic conditions^40^, whereas Fe-oxides (especially hematite) dominate in the red earth sediments of southern China owing to warm and seasonally dry conditions^10,41^. Hm/(Hm + Gt) ratios have been used as a paleo-environmental proxy in reconstructing Quaternary climate changes in the loess-paleosol sections of northern China and in other soil types^18,25,40^. However, previous paleoclimatic studies of red earth sediments in southern China have focused on other features, e.g., grain-size characteristics^3,42^, environmental magnetism^5,17^, geochemical compositions^43–45^, and clay-mineral assemblages^8,34,45^, rather than Hm/(Hm + Gt). The Hm/(Hm + Gt) ratios of soils are closely related to MAT and MAP^19^ and, thus, can effectively record paleoenvironmental changes during soil formation^18,22^.

High temperature and moisture levels contribute to weathering of tectosilicates (i.e., quartz and feldspars)^46,47^. Quartz is generally resistant under most conditions, whereas orthoclase and plagioclase are leached much more rapidly than quartz in an acidic environment^48^. Hence, the (orthoclase + plagioclase)/quartz ratio, or [(Or + Pl)/Q], can serve as a proxy for the degree of chemical weathering of soils^49^.

Magnetic susceptibility (χ_lf_) has been a useful proxy for reconstructing pedogenic histories and paleoclimatic changes in the loess–paleosol sequences of northern China^50–52^. However, χ_lf_ must be used cautiously as a paleoclimatic proxy in paleosols because of complications related to material sources, moisture regimes, pedogenic weathering intensity, and the presence of lithogenic ferrimagnetic minerals^53–56^. Especially in the red earth sediments of southern China, χ_lf_ does not accurately reflect paleoclimatic conditions because of the strong influence of post-depositional hydromorphic processes (i.e., related to the rise and fall of the groundwater table^57^). Although many ferromagnetic minerals contributing to the χ_lf_ signal are dissolved by such secondary processes, hematite and goethite are not significantly affected^30^. For this reason, the Hm/(Hm + Gt) ratio can be an effective paleo-environmental proxy in the red earth sections of southern China.

**Figure 8:**
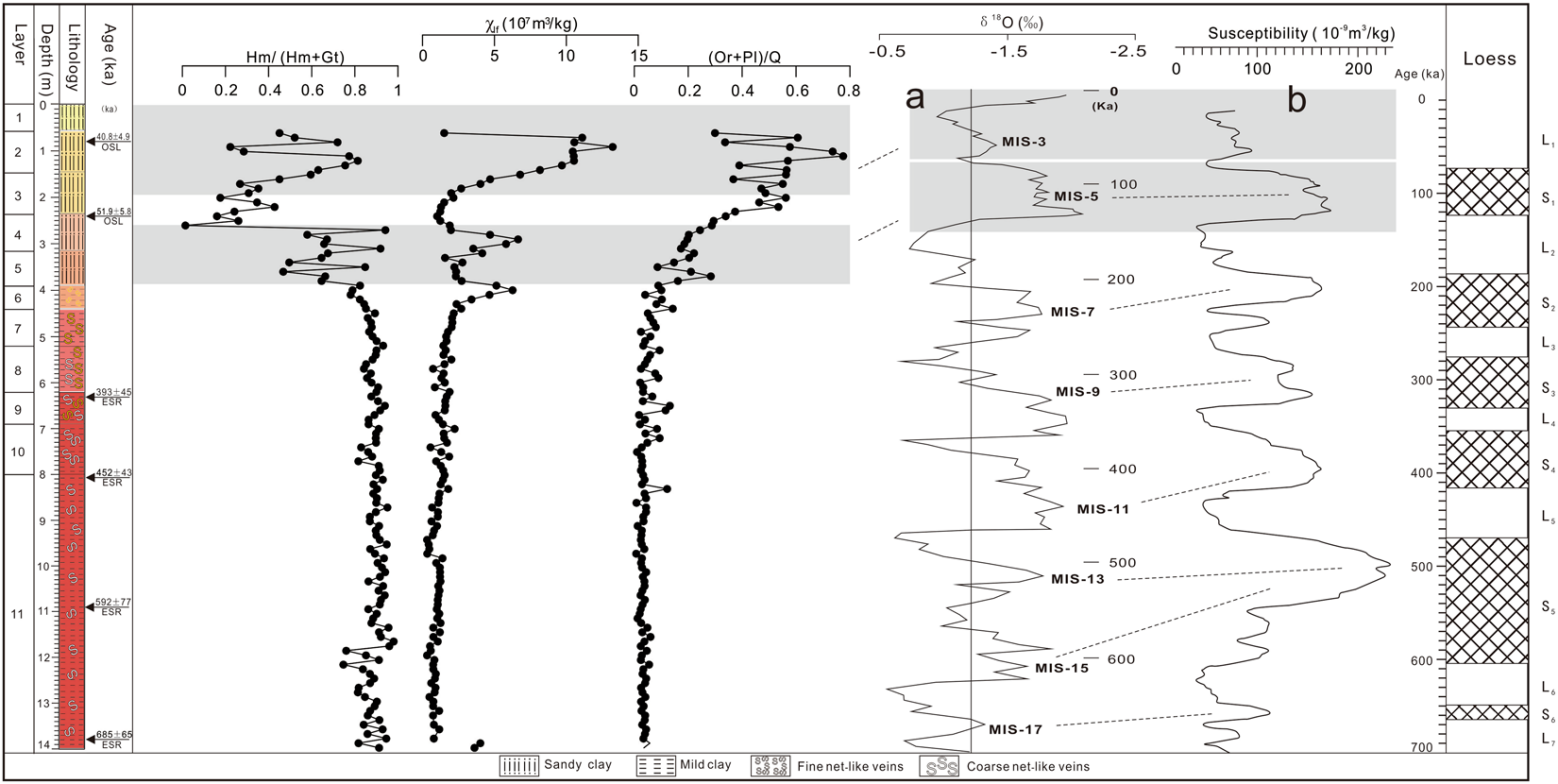
Comparison of proxies for Jiujiang red earth section with (**a**) δ^18^O records of the equatorial Pacific Core V28-238 (Shackleton and Opdyke, 1973) and (**b**) magnetic susceptibility from northern Chinese loess-paleosol sections (an average of data from Luochuan and Xifeng; Kukla *et al*.^56^). Ke Yin created this figure using Grapher 9 and CorelDRAW14.

Whereas the Hm/(Hm + Gt) and (Or + Pl)/Q profiles at Jiujiang are consistent in indicating a climate shift from warm/humid to cool/dry conditions during the middle to late Pleistocene, the magnetic susceptibility curve offers an apparently contradictory signal. In the loess-paleosol sections of northern China, higher χ_lf_ reflects greater neoformation of fine-grained pedogenic ferrimagnetic particles, which is generally interpreted to represent more highly weathered soils resulting from warmer and more humid climate conditions^25,50,58^. At Jiujiang, an upward increasing trend in χ_lf_ (Fig. 8) thus appears to contradict findings from the Hm/(Hm + Gt) and (Or + Pl)/Q profiles. However, the red earth sediments from southern China have experienced more intense chemical weathering than the loess deposits from northern China^2,30,45^, and the warmer, more humid conditions of southern China contributed to breakdown of ferrimagnetic particles^55^, resulting in lower χ_lf_ values. Hence, the paleoclimatic information recorded by the χ_lf_ proxy is, in fact, consistent with interpretations of the Hm/(Hm + Gt) and (Or + Pl)/Q data at Jiujiang. The relatively smooth upward changes in these proxies through the lower and middle units indicate that the study area experienced gradual climate change from warm and humid to cooler and drier during the middle to late Pleistocene, and the pronounced oscillations in all three proxies through the upper unit reflect multiple climate cycles since the last interglacial period^59–61^.

The existence of multiple climate subcycles with periodicities of ≤100 kyr during the middle and late Pleistocene has been demonstrated by magnetic susceptibility profiles from loess-paleosol sections in northern China (e.g., Luochuan and Xifeng) as well as by δ^18^O records from the equatorial Pacific (Fig. 8)^58,62^. Luochuan (Shanxi Province) and Xifeng (Gansu Province) are typical loess-paleosol sections of the Chinese Loess Plateau that are subdivided (from base to top) into the Wucheng Loess, Lower Lishi Loess, Upper Lishi Loess, Malan Loess, and Black Loam^58,62^. Although the Jiujiang section is stratigraphically equivalent to paleosols S_1_-S_6_ and loess units L_1_-L_7_ of the northern Chinese sections (Fig. 8), it does not show the same climate subcycles. The smooth pattern of climate change recorded by multiple proxies at Jiujiang between ~390 and 690 ka is due to the intense chemical weathering under warm, humid climate conditions experienced by this section. Consequently, the shorter-term (≤100-kyr) climate signals recorded in the loess-paleosol sections of northern China were overprinted in the red earth sections of southern China, which, thus, do not offer the same degree of paleoclimate detail. The controversies over paleoclimate interpretations of the red earth sediments of southern China are largely the result of use of different paleoclimate proxies in key studies^11,12^. The present study provides new insights into the implications of multiple proxies for understanding the paleoclimatic history of southern Chinese red earth sediments.

## Methods

**Figure 9:**
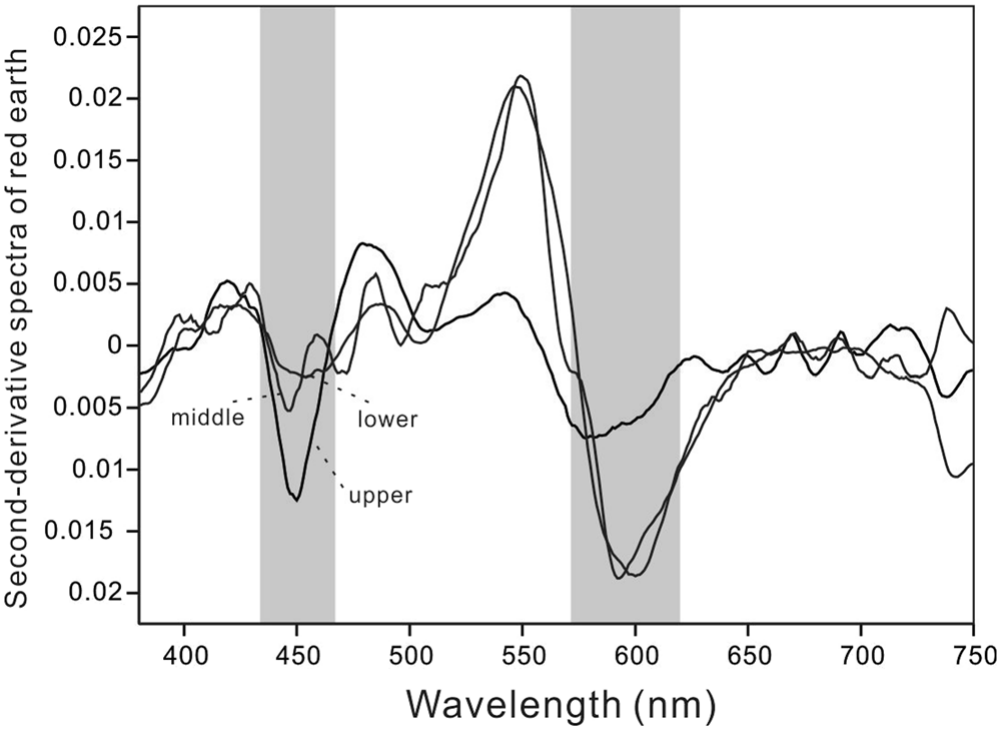
Second-derivative curves of representative samples from the upper, middle, and lower units of the Jiujiang red earth section. Ke Yin created this figure using Origin 8.0 and CorelDRAW14.

The Jiujiang section was sampled at 10 cm intervals. For diffuse reflectance spectrophotometry (DRS), the powders were pressed into black plastic holders at a pressure of >500 kPa. Reflectance spectra of samples were analyzed in a TU-1901 double-beam UV/VIS-spectrophotometer with a diffuse reflectance attachment (reflectance sphere) from the near-ultraviolet to the near-infrared (380–750 nm). Data processing was restricted to the visible spectrum (400–700 nm), which is the most sensitive region for Fe-oxide minerals with discrete measurements at 1-nm intervals^63^. The DRS method based on the second-derivative calculation was applied to quantify goethite and hematite via the band intensity in the second-derivative curves between the ~415-nm minimum and the ~445-nm maximum for goethite and between the ~535-nm minimum and the ~580-nm maximum for hematite^64^ (Fig. 9). The second-derivative method has an advantage over redness ratings in that it helps simultaneously to predict hematite and goethite contents, and it is also a fast, precise, and non-destructive method that has been widely used in many studies^18,64^. The concentrations of hematite and goethite in red earth samples were quantified using the second-derivative approach proposed by Scheinost *et al*.^64^ based on the following equations:

1 $${\rm{Hematite}}\,({\rm{g}}\,{{\rm{kg}}}^{-{\rm{1}}})=-{\rm{0.09}}+{{\rm{402Y}}}_{{\rm{1}}}({{\rm{r}}}_{{\rm{2}}}={\rm{0}}\mathrm{.85};\,{\rm{n}}=\mathrm{40};\,{\rm{p}}({\rm{\alpha }}) < {\rm{0.001}})$$

2 $${\rm{Goethite}}\,({\rm{g}}\,{{\rm{kg}}}^{-{\rm{1}}})=-{\rm{0.06}}+{{\rm{268Y}}}_{{\rm{2}}}({{\rm{r}}}_{{\rm{2}}}={\rm{0}}\mathrm{.86};\,{\rm{n}}=\mathrm{40};\,{\rm{p}}({\rm{\alpha }}) < {\rm{0.001}})$$

where Y_1_ stands for the second-derivative amplitude between the ~535-nm minimum and the ~580-nm maximum of hematite, and Y_2_ stands for the second-derivative amplitude between the ~415-nm minimum and the ~445-nm maximum of goethite.

For rock magnetic measurements, the dried powder samples were transferred to plastic boxes and subsequently compressed and fixed with cotton wool before securely fastening the lid in order to prevent movement of sediment particles during the measurements. The magnetic susceptibility was measured at room temperature with a MFK1-FA magnetic susceptibility meter (AGICO, Brno) and is given as mass-specific susceptibility (χ_lf_), using an alternating field of 200 A/m at 976 Hz measurement frequency. The susceptibility (χ_lf_) depends on the concentration and grain-size distribution of magnetic minerals^18^.

For X-ray diffraction analysis (XRD), bulk samples were air-dried and then crushed and ground manually to powder in an agate mortar with a pestle. The XRD patterns of the bulk samples were collected using a Panalytical X’Pert PRO DY2198 diffractometer at the Laboratory of Geological Process and Mineral Resources, China University of Geosciences (Wuhan). The instrument was operated at 40 kV and 40 mA with Ni-filtered Cu Kα radiation. It was measured from 3° to 35° 2θ at a scan rate of 4° 2θ/min and a step size of 0.02° 2θ. Relative abundances of quartz, plagioclase, and orthoclase were estimated semi-quantitatively using the weighting factor method with reference material of corundum^65^.

## References

[1] Hu XF (2010). Regional Distribution of the Quaternary Red Clay with Aeolian Dust Characteristics in Subtropical China and its Paleoclimatic Implications. Geoderma..

[2] Xiong S, Sun D, Ding Z (2002). Aeolian Origin of the Red Earth in Southeast China. Journal of Quaternary Science..

[3] Hu X, Zhu Y, Shen M (2005). Grain-Size Evidence for Multiple Origins of the Reticulate Red Clay in Southern China. Chinese Science Bulletin..

[4] Qiao Y (2003). Loess-Soil Sequences in Southern Anhui Province: Magnetostratigraphy and Paleoclimatic Significance. Chinese Science Bulletin..

[5] Zhao Q, Yang H (1995). A Preliminary Study On Red Earth and Changes of Quaternary Environment in South China. Quaternary Sciences..

[6] Zhu LD, Zhou SZ, Ye W, Hu X, Zhan XL (2005). Study On the Red Earth Sediment and Environmental Changes in South China. Journal of Zhejiang Normal University (Natural Sciences) (in Chinese with English abstracts)..

[7] Yin Q, Guo Z (2006). Mid-Pleistocene Vermiculated Red Soils in Southern China as an Indication of Unusually Strengthened East Asian Monsoon. Chinese Science Bulletin..

[8] Hong H, Gu Y, Yin K, Wang C, Li Z (2013). Clay Record of Climate Change Since the mid-Pleistocene in Jiujiang, South China. Boreas..

[9] Hu X (2015). Polypedogenic Case of Loess Overlying Red Clay as a Response to the Last Glacial–Interglacial Cycle in Mid-Subtropical Southeast China. Aeolian Research..

[10] Hong, H. *et al*. Geochemical Constraints On Provenance of the mid-Pleistocene Red Earth Sediments in Subtropical China. **290**, 97–108 (2013).

[11] Yang H, Xia WF, Zhao QG (1996). The Character of Magnetic Susceptibility of Red Earth Profile in South China and Palaeo-climateChanges. Acta Pedologica Sinica (in Chinese with English abstracts)..

[12] Yang H, Li XP, Zhao QG, Xia YF (1995). Characteristics of δ13C of Organic Matter in Xuancheng Eolian Sediment and Red Earth Series Profile. Acta Pedologica Sinica(in Chinese with English abstracts)..

[13] Li XS, Yang DY, Han HY (1998). A Preliminary Study On the Magnetic Susceptibility of Aeolian-Dust Deposition-Paleosol Sequence in the South of Anhui Province. Journal of Anhui Normal University (Natural Science) (in Chinese with English abstracts)..

[14] Li XS, Yang DY, Lu HY (1999). Oxide-Geochemistry Features and Paleoclimatic Record of the Aeolian-Dust Depositional Sequence in Southern Anhui. Marine Geology and Quaternary Geology (in Chinese with English abstracts)..

[15] Liu LW, Gong ZT (2000). Characteristics of Development of Quaternary Red Clay in Xuancheng, Anhui Province. Quaternary Sciences (in Chinese with English abstracts)..

[16] Liu LW, Gong ZT (2000). Development and Evolution of Red Paleosols. Marine Geology and Quaternary Geology (in Chinese with English abstract)..

[17] Hu X, Cheng T, Wu H (2003). Do Multiple Cycles of Aeolian Deposit-Pedogenesis Exist in the Reticulate Red Clay Sections in Southern China?. Chinese Science Bulletin..

[18] Buggle B (2014). Iron Mineralogical Proxies and Quaternary Climate Change in SE-European Loess-Paleosol Sequences. Catena..

[19] Cornell RM, Schwertmann U (2003). The Iron Oxides: Structure, Properties, Reactions, Occurrences and Uses.

[20] Ji J, Balsam W, Chen J, Liu L (2002). Rapid and Quantitative Measurement of Hematite and Goethite in the Chinese Loess-Paleosol Sequence by Diffuse Reflectance Spectroscopy. Clays and Clay Minerals..

[21] Nie J, Song Y, King JW, Fang X, Heil C (2010). HIRM Variations in the Chinese Red-Clay Sequence: Insights Into Pedogenesis in the Dust Source Area. Journal of Asian Earth Sciences..

[22] Post, D. F. *et al*. Correlations Between Field and Laboratory Measurements of Soil Color. In: Bigham, J. M., Ciolkosz, E. J. & Luxmoore, R. J. (Eds.), Soil Color. (SSSA Special Publications, 31, 35–49, 1993).

[23] Torrent, J. & Barrón, V. Laboratory Measurement of Soil Color: Theory and Practice. In: Bigham, J. M., Ciolkosz, E. J. & Luxmoore, R. J. (Eds), Soil Color. (SSSA Special Publications, 31, Pp. 21–34, 1993).

[24] Torrent J, Liu Q, Bloemendal J, Barrón V (2007). Magnetic Enhancement and Iron Oxides in the Upper Luochuan Loess–Paleosol Sequence, Chinese Loess Plateau. Soil Science Society of America Journal..

[25] Torrent, J. & Barrón, V. Diffuse Reflectance Spectroscopy of Iron Oxides. In: Hubbard, a.T. (Ed.), Encyclopedia of Surface and Colloid Science, Vol. 1, Marcel Dekker, New York, Pp. 1438–1446. (2002).

[26] Panaiotu CG, Panaiotu EC, Grama A, Necula C (2001). Paleoclimatic Record From a Loess-Paleosol Profile in Southeastern Romania. . Physics & Chemistry of the Earth Parts(A).

[27] Liu Q, Deng C, Torrent J, Zhu R (2007). Review of Recent Developments in Mineral Magnetism of the Chinese Loess. Quaternary Science Reviews..

[28] Torrent J, Liu QS, Barrón V (2010). Magnetic Susceptibility Changes in Relation to Pedogenesis in a Xeralf Chronosequence in Northwestern Spain. European Journal of Soil Science..

[29] Hu XF, Wei J, Xu LF, Zhang GL, Zhang WG (2009). Magnetic Susceptibility of the Quaternary Red Clay in Subtropical China and its Paleoenvironmental Implications. Palaeogeography, Palaeoclimatology, Palaeoecology..

[30] Liu C, Deng C, Liu Q (2012). Mineral Magnetic Studies of the Vermiculated Red Soils in Southeast China and their Paleoclimatic Significance. Palaeogeography, Palaeoclimatology, Palaeoecology..

[31] Liu QS (2010). Environmental Magnetic Study of a Xeralf Chronosequence in Northwestern Spain: Indications for Pedogenesis. Palaeogeography, Palaeoclimatology, Palaeoecology..

[32] Vidic NJ, Singer MJ, Verosub KL (2004). Duration Dependence of Magnetic Susceptibility Enhancement in the Chinese Loess–Palaeosols of the Past 620 Ky. Palaeogeography Palaeoclimatology Palaeoecology..

[33] Hong H, Churchman GJ, Gu Y, Yin K, Wang C (2012). Kaolinite–Smectite Mixed-Layer Clays in the Jiujiang Red Soils and their Climate Significance. Geoderma..

[34] Yin K (2014). Characterisation of the Hydroxy-Interlayered Vermiculite From the Weathering of Illite in Jiujiang Red Earth Sediments. Soil Research..

[35] Yin K (2013). Hydroxy-Interlayered Vermiculite Genesis in Jiujiang late-Pleistocene Red Earth Sediments and Significance to Climate. Applied Clay Science..

[36] Wada K, Kakuto Y (1983). Intergradient Vermiculite-Kaolin Mineral in a Korean Ultisol. Clays and Clay Minerals..

[37] Hirai H, Araki S, Kyuma K (1989). Clay Mineralogical Properties of Brown Forest Soils in Northern Kyoto with Special Reference to their Pedogenetic Process. Soil Science and Plant Nutrition..

[38] Vicente MA, Elsass F, Molina E, Robert M (1997). Palaeoweathering in Slates From the Iberian Hercynian Massif (Spain); Investigation by TEM of Clay Mineral Signatures. Clay Minerals..

[39] Ji J (2004). High Resolution Hematite/Goethite Records From Chinese Loess Sequences for the Last Glacial‐Interglacial Cycle: Rapid Climatic Response of the East Asian Monsoon to the Tropical Pacific. Geophysical Research Letters.

[40] Zhang H, Han Y, Xing G (1989). Study On the Iron Oxides for Torrid Red Earths by Using the Mossbauer Spectroscopy. Acta Pedologica Sinica (in Chinese with English abstracts)..

[41] Li XS, Yang DY, Lu HY, Han HY (1997). The Grain-Size Features of Quaternary Aeolian-Dust Deposition Sequence in South Anhui and their Significance. Marine Geology and Quaternary Geology (in Chinese with English abstracts)..

[42] Yang SY, Li CX, Yang DY, Li XS (2004). Chemical Weathering of the Loess Deposits in the Lower Changjiang Valley, China, and Paleoclimatic Implications. Quaternary International..

[43] Chen Y (2008). Chemical Weathering Intensity and Element Migration Features of the Xiashu Loess Profile in Zhenjiang, Jiangsu Province. Journal of Geographical Sciences..

[44] Hong H, Gu Y, Li R, Zhang K, Li Z (2009). Clay Mineralogy and Geochemistry and their Palaeoclimatic Interpretation of the Pleistocene Deposits in the Xuancheng Section, Southern China. Journal of Quaternary Science..

[45] White AF, Blum AE (1995). Effects of Climate On Chemical_ Weathering in Watersheds. Geochimica et Cosmochimica Acta..

[46] Brady PV, Carroll SA (1994). Direct Effects of CO_2_ and Temperature On Silicate Weathering: Possible Implications for Climate Control. Geochimica et Cosmochimica Acta..

[47] Pettijohn, F. J., Potter, P. E. & Siever, R. Sand and Sandstone: New York, Springer-Verlag, 618 P.426 (1973).

[48] Nesbitt HW, Fedo CM, Young GM (1997). Quartz and Feldspar Stability, Steady and Non‐Steady-State Weathering, and Petrogenesis of Siliciclastic Sands and Muds. The Journal of Geology..

[49] Liu T (1985). Loess and the Environment.

[50] An Z, Kukla GJ, Porter SC, Xiao J (1991). Magnetic Susceptibility Evidence of Monsoon Variation On the Loess Plateau of Central China During the Last 130,000 Years. Quaternary Research..

[51] Maher BA, Thompson R (1995). Paleorainfall Reconstructions from Pedogenic Magnetic Susceptibility Variations in the Chinese Loess and Paleosols. Quaternary Research..

[52] Sun J, Liu T (2000). Multiple Origins and Interpretations of the Magnetic Susceptibility Signal in Chinese Wind-Blown Sediments. Earth & Planetary Science Letters..

[53] Virina EI, Faustov SS, Heller F (2000). Magnetism of Loess-Palaeosol Formations in Relation to Soil-Forming and Sedimentary Processes. Physics & Chemistry of the Earth Part A Solid Earth & Geodesy..

[54] Guo B, Zhu RX, Roberts AP, Florindo F (2001). Lack of Correlation Between Paleoprecipitation and Magnetic Susceptibility of Chinese Loess/Paleosol Sequences. Geophysical Research Letters..

[55] Bloemendal J, Liu XR (2005). Magnetism and Geochemistry of Two Plio-Pleistocene Chinese Loess-Palaeosol Sequences-Implications for Quantitative Palaeoprecipitation Reconstruction. Palaeogeography, Palaeoclimatology, Palaeoecology..

[56] Kukla G, An ZS, Melice JL (1990). Magnetic Susceptibility Record of Chinese Loess. Transactions of the Royal Society of Edinburgh: Earth Sciences..

[57] Hong H, Gu Y, Yin K, Zhang K, Li Z (2010). Red Soils with White Net-Like Veins and their Climate Significance in South China. Geoderma..

[58] Heinrich H (1988). Origin and Consequences of Cyclic Ice Rafting in the Northeast Atlantic Ocean During the Past 130,000 Years. Quaternary Research..

[59] Dansgaard W (1993). Evidence for General Instability of Past Climate From a 250-Kyr Ice-Core Record. Nature..

[60] Thouveny N (1994). Climate Variations in Europe Over the Past 140 Kyr Deduced From Rock Magnetism. Nature..

[61] Kukla G, An Z (1989). Loess stratigraphy in central China. Palaeogeography, Palaeoclimatology, Palaeoecology..

[62] Shackleton NJ, Shackleton NJ, Opdyke ND (1973). Oxygen Isotope and Paleomagnetic Stratigraphy of Equatorial Pacific Core V28-238: Oxygen Isotope Temperatures and Ice Volumes On 106 Yr Scale. Quaternary Research..

[63] Deaton BC, Balsam WL (1991). Visible Spectroscopy; A Rapid Method for Determining Hematite and Goethite Concentration in Geological Materials. Journal of Sedimentary Research..

[64] Scheinost AC, Chavernas A, Barron V, Torrent J (1998). Use and Limitations of Second-Derivative Diffuse Reflectance Spectroscopy in the Visible to Near-Infrared Range to Identify and Quantity Fe Oxide Minerals in Soils. Clays and Clay Minerals..

[65] Hillier S (2000). Accurate Quantitative Analysis of Clay and Other Minerals in Sandstones by XRD: Comparison of a Rietveld and a Reference Intensity Ratio (RIR) Method and the Importance of Sample Preparation. Clay Minerals..

